# Genome Editing Using CRISPR-Cas9 and Autoimmune Diseases: A Comprehensive Review

**DOI:** 10.3390/ijms23031337

**Published:** 2022-01-25

**Authors:** Min Ho Lee, Jae Il Shin, Jae Won Yang, Keum Hwa Lee, Do Hyeon Cha, Jun Beom Hong, Yeoeun Park, Eugene Choi, Kalthoum Tizaoui, Ai Koyanagi, Louis Jacob, Seoyeon Park, Ji Hong Kim, Lee Smith

**Affiliations:** 1 Yonsei University College of Medicine, Seoul 03722, Korea; mhlee164@naver.com (M.H.L.); eric1@kaist.ac.kr (D.H.C.); bigjbh2@naver.com (J.B.H.); yeoeun@yonsei.ac.kr (Y.P.); 4606347@naver.com (E.C.); harryme1713@yonsei.ac.kr (S.P.); 2 Department of Pediatrics, Yonsei University College of Medicine, Seoul 03722, Korea; shinji@yuhs.ac (J.I.S.); AZSAGM@yuhs.ac (K.H.L.); 3 Department of Nephrology, Yonsei University Wonju College of Medicine, Wonju 26426, Korea; kidney74@yonsei.ac.kr; 4 Korea Advanced Institute for Science and Technology, Graduate School of Medical Science and Engineering, Daejeon 34141, Korea; 5 Laboratory Microorganismes and Active Biomolecules, Sciences Faculty of Tunis, University Tunis El Manar, Tunis 1068, Tunisia; kalttizaoui@gmail.com; 6 Parc Sanitari Sant Joan de Deu/CIBERSAM, Universitat de Barcelona, Fundacio Sant Joan de Deu, Sant Boi de Llobregat, 08830 Barcelona, Spain; a.koyanagi@pssjd.org (A.K.); louis.jacob.contacts@gmail.com (L.J.); 7 ICREA, Pg. LluisCompanys 23, 08010 Barcelona, Spain; 8 Faculty of Medicine, University of Versailles Saint-Quentin-en-Yvelines, 78180 Montigny-le-Bretonneux, France; 9 Department of Pediatrics, Gangnam Severance Hospital, Yonsei University College of Medicine, Seoul 06273, Korea; 10 Centre for Health, Performance, and Wellbeing, Anglia Ruskin University, Cambridge CB1 1PT, UK; Lee.Smith@aru.ac.uk

**Keywords:** CRISPR-Cas9, genome editing, autoimmune diseases

## Abstract

Autoimmune diseases are disorders that destruct or disrupt the body’s own tissues by its own immune system. Several studies have revealed that polymorphisms of multiple genes are involved in autoimmune diseases. Meanwhile, gene therapy has become a promising approach in autoimmune diseases, and clustered regularly interspaced palindromic repeats and CRISPR-associated protein 9 (CRISPR-Cas9) has become one of the most prominent methods. It has been shown that CRISPR-Cas9 can be applied to knock out proprotein convertase subtilisin/kexin type 9 (PCSK9) or block PCSK9, resulting in lowering low-density lipoprotein cholesterol. In other studies, it can be used to treat rare diseases such as ornithine transcarbamylase (OTC) deficiency and hereditary tyrosinemia. However, few studies on the treatment of autoimmune disease using CRISPR-Cas9 have been reported so far. In this review, we highlight the current and potential use of CRISPR-Cas9 in the management of autoimmune diseases. We summarize the potential target genes for immunomodulation using CRISPR-Cas9 in autoimmune diseases including rheumatoid arthritis (RA), inflammatory bowel diseases (IBD), systemic lupus erythematosus (SLE), multiple sclerosis (MS), type 1 diabetes mellitus (DM), psoriasis, and type 1 coeliac disease. This article will give a new perspective on understanding the use of CRISPR-Cas9 in autoimmune diseases not only through animal models but also in human models. Emerging approaches to investigate the potential target genes for CRISPR-Cas9 treatment may be promising for the tailored immunomodulation of some autoimmune diseases in the near future.

## 1. Introduction

Autoimmune diseases are disorders that destructs or disrupts the body’s own tissues by its own immune system [1]. Normally, the body’s immune system has immunologic tolerance so that it does not hurt its own body. However, in an autoimmune disease, the immune system attacks its own tissues and organs. Autoimmune diseases occur in up to 3–5% of the general population [1], and they decrease life expectancy. It has been shown that autoimmune diseases are associated with mood disorders [2] and impaired quality of life. Additionally, it has been known that various genetic factors account for autoimmune diseases, such as rheumatoid arthritis, systemic lupus erythematosus, type 1 diabetes mellitus, and multiple sclerosis [3].

**Figure 1:**
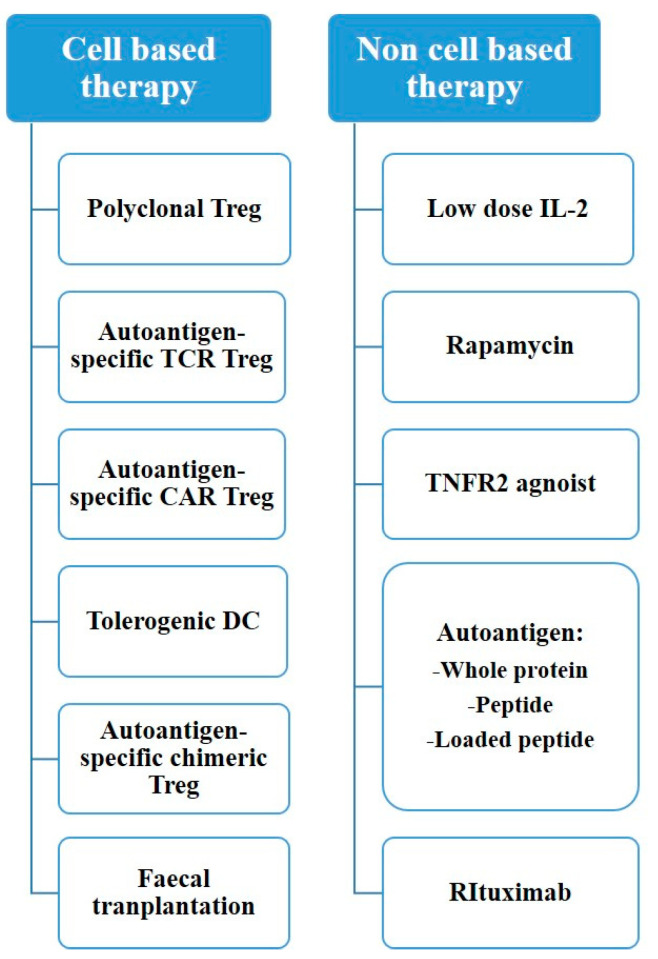
Treatment options for autoimmune disease.

**Figure 2:**
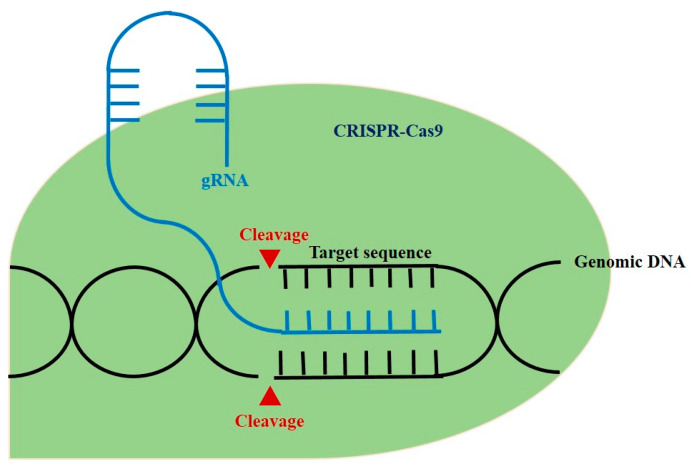
The schematic diagram of CRISPR-Cas 9 in genomic engineering.

Meanwhile, gene therapy has become a promising approach as our understanding of the immunological and molecular basis of autoimmune diseases advances [4]. The ultimate objective of gene therapy is regulation of the level of inflammatory cytokines and infiltration of lymphocytes to the affected sites (Figure 1) [4]. There has been a growing need for gene-editing therapeutic approaches, with clustered regularly interspaced palindromic repeats and CRISPR-associated protein 9 (CRIPSR-Cas9) being one of the most prominent methods [5]. CRISPR-Cas9, which has been widely renowned as genome-editing technology, is originally an adaptive immune system of bacteria and archaea and can be applicable in eukaryotic cells with single guide RNA (sgRNA) that contains complementary base sequence of a gene of interest [6]. By creating a synthetic sgRNA of a sequence complementary to a particular gene, Cas9, an RNA-guided DNA nuclease can knock out the gene (Figure 2) [7]. Gene knockout occurs during repair through non-homologous end joining (NHEJ) responding to the double strand breakage. In addition, knock-in can be performed via homologous direct recombination (HDR) if adequate template DNA is present [7,8]. The possibility of therapeutic application of CRISPR-Cas9 has been emerging based on various studies ranging from gene-editing in embryo with germline genetic variant to in vivo delivery in animal models of specific genetic diseases using viral or lipid vectors [5].

Application of CRISPR-Cas9 in genetic diseases has been reported in several investigations [9,10,11,12,13,14,15]. CRISPR-Cas9 can be applied to knock out proprotein convertase subtilisinkexin type 9 (PCSK9) or block PCSK9, resulting in lowering low-density lipoprotein cholesterol [9,10]. Possibilities are not limited to frequently occurring diseases, and it holds great promise to treat rare diseases such as Ornithinetranscarbamylase (OTC) deficiency and hereditary tyrosinemia [11,12]. However, limited studies on the treatment of autoimmune disease using CRISPR-Cas9 have been reported so far. In this review, we highlight the current and potential use of CRISPR-Cas9 in the management of autoimmune diseases.

## 2. CRISPR-Cas9 and Autoimmune Diseases

### 2.1. In Vivo Therapeutic Trials Using CRISPR-Cas9

Autoimmunity usually occurs through systemic immune reaction caused by the failure to regulate the immune response adequately. For in vivo genetic correction via CRISPR-Cas9, sgRNA and Cas9 must be penetrated into the nucleus of the cell to edit the targeted DNA site. There are two major CRISPR-Cas9 delivery systems: viral vectors and lipid nanoparticles [5]. When using the viral transfer vector, adeno-associated virus (AAV), which has low possibility of genomic integration into foreign genes and has been demonstrated to be safe, is most widely used [16]. Given that AAV is small in size, large-sized SpCas9 derived from *Streptococcus pyogenes* is infrequently used despite its high efficacy, thus smaller Cas9 proteins derived from other bacterial species are used [17]. Cas9 derived from *Staphylococcus aureus* (SaCas9) or *Campylobacter jejuni* (CjCas9) can be packed together with sgRNA in AAV and applied to in vivo gene correction with a decent efficiency [18,19].

PCSK9, which regulates low-density lipoprotein (LDL) and is known as a major risk factor for atherosclerosis and coronary heart disease, was knocked out in hepatocytes using NHEJ with SaCas9, resulting in a significant decrease in LDL [20]. Another study reported that in vivo delivery of meganuclease with AAV targeting PCSK9 showed reduced levels of serum cholesterol [9,10]. Rare metabolic diseases have also been studied. A mouse model of OTC deficiency was treated with AAV8 and SaCas9, and a limited but significant therapeutic effect was observed in the management of hereditary tyrosinemia [11,12]. In some studies of Duchenne muscular dystrophy (DMD), the pathogenic mutation within exon23 was knocked out, and improvement in skeletal muscle and myocardial function was observed [13,14,15].

### 2.2. Inflammatory Molecules, Immunogenetics and CRISPR-Cas9

Autoimmunity is based on the simple concept of an imbalance between pro-inflammatory and anti-inflammatory stimuli, leading to an abnormal immune response. Cytokines like interleukin-1 (IL-1) and tumor necrosis factor (TNF) play an important role in inflammation, thus reducing the expression of these cytokines eventually leads to abrogation of the autoimmune process [21]. For example, interleukin-36 (IL-36) cytokines (IL-36A, IL-36B, and IL-36G) and members of the IL-1 family can cause pro-inflammatory effects in the skin and other organs [22]. Studies have shown that cytokines belonging to the IL-36 family may play an important role in the development of autoimmune diseases such as rheumatoid arthritis (RA), systemic lupus erythematosus (SLE), inflammatory bowel disease (IBD), Sjögren’s syndrome, and psoriasis vulgaris [23,24,25]. Myeloid differentiation primary response gene 88 (*MyD88*) adapter protein is known to be activated by IL-36 stimulation. Inactivation of *MyD88* adapter protein by CRISPR-Cas9 reduced the activity of differentially expressed genes inducing the expression of IL-1B and IL-36G [26].

Tumor necrosis factor alpha-induced protein 3 (*TNFAIP3*) is induced by TNF-alpha and is known to inhibit NF-kappa B activation and TNF-mediated apoptosis [27]. The genetic variation of *TNFAIP3* has been reported to be associated with susceptibility to develop SLE and RA [28,29]. In SLE, a genome-wide association study demonstrated that UBE2L3 could be a novel therapeutic target. *UBE2L3* was identified as the key E2 enzyme for linear ubiquitin chain assembly complex (LUBAC) and essential for LUBAC-mediated activation of NF-kB [30]. In addition, association with familial Behçet-like auto inflammatory syndrome and infantile-onset intractable IBD was also reported [31,32]. A study on transcription activation-like effector nuclease (TALEN)-mediated knockout of *TNFAIP3* demonstrated that correcting pathogenic *TNFAIP3* variants can possibly reverse its related autoimmune phenotypes [33]. In addition, knockout of a candidate causal variant, rs6927172, by CRISPR-Cas9 gene editing influenced the expression of the IL-20RA and *TNFAIP3* genes, potentially involved in the autoimmune response [34].

In addition to cytokines, many genetic variants that contribute to the development of autoimmune diseases are associated with T cells. For example, FoxP3 + regulatory T cells play an important role in immunological tolerance [35]. Immuno-dysregulation polyendocrinopathy, enteropathy X-linked (IPEX) syndrome caused by mutation of FoxP3 in humans can lead to a variety of autoimmune disorders in early childhood [36]. In IPEX, FoxP3 is decreased and the function of Tregs is remarkably degraded. In fact, epigenetic editing of the promoter and conserved non-coding sequence 2(CNS2) in the FoxP3 gene showed a 20–30% reduction in the effector T cell division [37] and thus CRISPR-Cas9 may be a potential therapeutic approach to manage IPEX cases.

### 2.3. CRISPR-Cas9 and iPSC

In autoimmunity, various treatment methods have been studied using CRISPR-Cas9. Induced pluripotent stem cells (iPSCs) were produced by adding interleukin-1 receptor antagonist (IL1Ra) or soluble TNF receptor gene by using CRISPR-Cas9 gene editing and differentiated into articular cartilage. This showed that inflammatory response was attenuated by initiating dynamic negative feedback upon stimulation with inflammatory cytokines [38]. Other studies have reported that articular cartilage is resistant to IL-1alpha-mediated tissue degradation by the deletion of interleukin-1 receptor 1 (IL1r1) in murine iPSCs [39].

Gene editing in mesenchymal stem cells (MSCs) was utilized with CRISPR-Cas9 to target the endogenous activation of pancreatic transcription factors and MSC chemokine receptors. MSCs were differentiated into surrogate insulin-producing cells and may be transplanted through ex vivo expansion and transplantation while maintaining their immunomodulatory properties [40]. Other studies have successfully activated endogenous human insulin transcription using sgRNAs targeting multiple insulin promoter and a nuclease-deficient Cas9-virion protein 160 gene (dCas9-VP160) in human embryonic kidney 293 T cell (HEK293T), Hela, and human fibroblasts [41].

## 3. CRISPR-Cas9 and Rheumatoid Arthritis

Three studies tested genetic therapy in human cell models in RA. From these studies, the *MYC*, *FOXO1* gene, SNP rs6927172, *TNFAIP3*, *OLIG3* gene, and *miR-155* have been suggested as appropriate candidates for CRISPR-Cas9 treatment.

Yang et al. suggested that *MYC* and *FOXO1* genes are associated with rheumatoid arthritis [42]. It has been thought that in RA patients, CD4+ T-cells express higher autophagy, and *MYC* was thought to be the regulator of the pathway [42]. *FOXO1* has also been thought to be correlated with RA activity [42]. This study, by collecting ATAC-seq, Hi-C, Capture Hi-C, and nuclear RNA-seq data in stimulated CD4+ T cells over 24 hours, provides evidence that *MYC* and *FOXO1* genes may be causal factors of RA [42]. In the study by Yu et al., an intergenic SNP rs6927172 on chromosome 6q23 region was found to be associated with RA disease course according to a genome-wide association study [43]. According to the study, many genes such as *IL20RA*, *IL22RA2*, *IFNGR1*, *OLIG3*, and *TNFAIP3* flank the SNP area, and when they disrupt the SNP area using CRISPR-Cas9, only *TNFAIP3* and *OLIG3* showed decreased expressivity [43]. It shows that the SNP rs6927172, *TNFAIP3* and *OLIG3* are importantly associated with RA disease course [43]. Jing et al. demonstrated that microRNA 155 (*miR-155*) could be an important pro-inflammatory factor in RA patients [44]. They used an *miR-155* knockout RAW 264.7 macrophage cell line and found that in the cell line, SHP1 was up-regulated and pro-inflammatory cytokine making process was impaired [44]. Thus, they suggest that genome editing of *miR-155* can be a potential therapeutic strategy for RA [44].

## 4. CRISPR-Cas9 and Inflammatory Bowel Disease

Four studies in human cell models and another four studies in mouse models tested genetic therapy in IBD. From these studies, *JAK2*, *TL1A*, *SGK2*, *PTPN2*, *c-MYC*, *HDAC7*, and IFN- γ genes and miR-125a have been suggested as appropriate candidates for CRISPR-Cas9 treatment.

### 4.1. Studies on CRISPR-Cas9 and IBD Using Human Immune Cells

Analysis of rs1887428, located in the promoter region of the Janus kinase 2 [*JAK2*] gene, by Cardinale et al. revealed target genes affected by this polymorphism [45]. Authors found out that the risk allele of rs1887428 is bound by the transcription factor (TF) RBPJ [45]. According to the paper, rs1887428 did not have very large impacts on JAK2 expression but its impact was on *STAT5B* amplified downwards [45]. The limitation of this study was that it could not find the TF binding motif and expression quantitative trait locus [45]. Mokhtar et al. demonstrated that the expression of *SGK2* part of the PI3K/Akt pathway affects IBD disease course [46]. Immunohistochemistry was performed on SW480 cells and *SGK2* gene knockdown was performed by CRISPR gRNA [46]. As a result, SGK2 protein was localized in the cytoplasm of colonic epithelial cells both in long and short duration ulcerative colitis [46]. This indicates that *SGK2* gene may have a crucial role in IBD disease course [46]. In the study by Li et al., *PTPN2* gene variants occurred in Crohn’s disease patients [46]. In these patients, *PTPN2* had loss of function mutation and it was associated with rs7234029 SNP [46]. They used subepithelial myofibroblast cells (SEMF) [46]. The result showed that *PTPN2* expression was increased in the affected ileum compared to normal ileum and, in contrast, CRISPR-Cas9 mediated *PTPN2* deletion resulted in higher levels of *STAT3* and Erk1/2 phosphorylation and proliferation [47]. It indicates that *PTPN2* gene variants and SNP rs7234029 have a crucial role in Crohn’s disease [47]. Matthews et al. investigated rs6651252 SNP on chromosome 8 [48]. The study demonstrated that rs6651252 resides within a Wnt responsive DNA enhancer element and disease-associated allele increases binding of the *TCF7L2* transcription factor to the region [48]. Using CRISPR-Cas9, they found that rs6651252 enhancer regulates expression of the *c-MYC*, and they revealed that *MYC* expression levels are elevated in the Crohn’s disease patients [48].

### 4.2. Studies on CRISPR-Cas9 and IBD Using Non-Human Cells

Pai et al. focused on a tumor necrosis factor super family member TL1A [49]. According to the study, spontaneous ileitis was present in TL1A transgenic mice [49]. When neutralizing anti-TL1A was used, brush border fanning and bacterial endocytosis caused by MLCK reduced [49]. Lastly, the expression of TL1A, IFNγ, and both MLCK1 and 2 was upregulated in the mucosa of IBD patients. [48] Thus, the study demonstrates that the flare-up of TL1A, IFNγ, and MLCK is associated with IBD diseases course [49]. Friedrich et al. used primary colonic epithelial cells (CEC), human T84, and murine CMT93 to characterize functions of *HDAC7* [50]. Knockout mice were generated using CRISPR-Cas9 system [50]. The result showed that *HDAC* function was reduced in IBD patients, and according to the knockout mice, *HDAC* was found to play a crucial role in maintenance of the intestinal barrier [50]. Eftychi et al. crossed NEMOIEC-KO with IFNγ −/− mice generated by CRISPR-Cas9 to demonstrate that IFN-γ can have a crucial role in colon inflammation [51]. They found that many pro-inflammatory cytokines such as Tnf, IL1b, and IL6 showed decreased expressivity in the knockout mice and suggested that IFN-γ is essential for colon inflammation [51]. Ge et al. showed that microRNA-125a can suppress intestinal mucosal inflammation [52]. According to their results, miR-125a expression was reduced in mucosa and peripheral blood of IBD patients [52]. MiR-125a suppresses Th1/Th17 cell differentiation and TNF-α production. In addition, miR-125a knockout mice showed more severe forms of colitis [52].

## 5. CRISPR-Cas9 and Systemic Lupus Erythematosus

Two studies tested genetic therapy in human cell models in SLE. From these studies, the A20 DUB and *CXorf21* gene have been suggested as appropriate candidates for CRISPR-Cas9 treatment. Odqvist et al. aimed to assess whether *TNFAIP3* (A20) deubiquitinase (DUB) increases the risk of SLE [53]. They used CRISPR-Cas9 to make human U937 monocytes with A20 DUB inactivating *C103A* knock-in mutation [53]. Results showed that A20 *C103A* cells or cells with rs2230926 polymorphism drew increased neutrophil extracellular trap and increased frequency of auto antibodies to citrullinated epitopes [53]. They concluded that interrupting the A20 DUB domain increases susceptibility to SLE [53]. Harris et al. focused on the involvement of Chromosome X open reading frame 21 (*CXorf21*) genes in SLE disease course [54]. They conducted in vitro CRISPR-Cas9 knockdown experiments and found that *CXorf21* knockdown resulted in a decreased expression of TNF-alpha and IL-6 [54]. They concluded that sexually dimorphic expression of *CXorf21* could be a risk factor for SLE [54].

## 6. CRISPR-Cas9 and Multiple Sclerosis

Four studies tested genetic therapy in human cell models in MS. From these studies, the *IR7R* gene, the RNA helicase DEAD box polypeptide 39B, the IL2RA gene, and the *TNFRSF1A* gene have been suggested as appropriate candidates for CRISPR-Cas9 treatment. Gregory et al. demonstrated that the IL7Rα-related immune response pathway is crucial in the pathogenesis of MS [55]. The rs6897932 SNP in exon 6 of IL7R affects gene expression of the soluble versus membrane-bound form of the protein and increases the risk of MS [55]. The soluble form of IL7Rα is a driver of increased MS risk. The ‘C’ allele of rs6897932 is associated with MS, and it enhances skipping of exon 6 of the IL7R gene by augmenting an exon splicing silencer [55]. The finding suggests that rs6897932 may affect the amount of the soluble versus membrane-bound form of the protein [55]. Isoforms are significant to regulate IL7 signaling pathway [55]. As a result, there is a direct relationship between alternative rs6897932 alleles and MS risk. Galarza-Munoz et al. demonstrated that human epistatic interaction is associated with MS risk. RNA helicase *DDX39B*, which is a potent activator of IL7R exon 6 and repressor of the soluble form of IL7R, strongly correlates with MS risk [56]. It was shown that epistatic interaction between rs2523506 in *DDX39B* and rs6897932 in IL7R regulates IL7R exon 6 splicing and increases risk of MS [56]. Not only local mutations in the IL7R gene, but also genetic and functional epistasis with IR7R gene is related to the risk of MS. In another study, Maier et al. showed that the IL-2RA gene has many variants that increase the risk of MS, and several variants were associated with sIL-2RA levels independently [57]. Maier et al. investigated IL-2RA genetic heterogeneity in MS and type 1 diabetes mellitus (T1DM) together, which are related to share alleles. IL2RA variants contributed to the risk of MS and T1DM respectively [57]. rs2104286 SNP located in intron 1 of IL2RA was mainly associated with susceptibility of MS [56]. Gregory et al. showed that TNF blocking agents are effective treatment options for non-MS autoimmune diseases. The investigation suggests that rs1800693 SNP in the *TNFRSF1A* gene, which encodes tumor necrosis factor receptor 1 (TNFR1), is associated with MS as the causal variant [58]. TNF blocking agents have side effects that promote the onset of MS and other autoimmune diseases such as RA. However, the study by Gregory et al. reported a disease signal for rs1800693 predictive of side effects [58].

## 7. CRISPR-Cas9 and Type 1 Diabetes Mellitus

One study tested genetic therapy in human cell models in Type 1 Diabetes mellitus (DM), and the other study tested this in mice models. From these studies, the AID/RAD51, and SNP rs10914542 of LCK gene have been suggested as appropriate candidates for CRISPR-Cas9 treatment. Zhu et al. attempted to reveal the role of SNPs of the Lymphocyte-specific protein tyrosine kinase (LCK) gene [59]. They gained blood samples from type 1 DM patients and used CRISPR-Cas9 to find the role of LCK SNP [59]. Among SNPs, only SNP rs10914542 showed meaningful association [59]. Thus, they demonstrate that G allele of rs10914542 SNP in LCK increases the risk of Type 1 DM [59]. Jeremy et al. used activation-induced cytidine deaminase (AID) gene knockout mice and found that AID/RAD51 could be a target for treatment of Type 1 DM patients [60].

## 8. CRISPR-Cas9 and Psoriasis

One study tested genetic therapy in human cell models in psoriasis and the other study tested this in mouse models. From these studies, desmoglein 1 and ERAP1 genes have been suggested as appropriate candidates for CRISPR-Cas9 treatment. Arakawa et al. demonstrated that ERAP1 has a crucial role in the development of psoriasis [61]. They generated ERAP1 knockout melanoma cell line and revealed that epistasis between HLA-C*06:02 and ERAP1 variants affects psoriasis [61]. The course includes ADAMTS-like protein 5 (ADAMTSL5) and immunogenicity of melanocytes [61]. Roth-Carter et al. used desmoglein 1 knockout mice and found that the knockout mice exhibited barrier impairment [62]. Analysis of E18.5 skin of knockout mice revealed that inhibition of Dsg1 leads to increase in pathways to psoriatic process [62]. These results support a role of Dsg1 for differentiation of epidermis, formation of barriers, and regulation of inflammatory responses [62].

## 9. CRISPR-Cas9 and Type 1 Coeliac Disease

One study tested genetic therapy in cell models regarding coeliac disease [63]. From the study, the α- or γ-gliadin genes have been suggested as appropriate candidates for CRISPR-Cas9 treatment. According to Jouanin et al., wheat grains contain gluten proteins in which immunogenic epitopes can trigger coeliac disease [63]. They analyzed α- and γ-gliadin gene sequences and generated CRISPR-Cas9 constructs targeting α- or γ-gliadins [63]. The result showed that it is possible to use CRISPR-Cas9 to edit α- or γ-gliadins and make safe forms of grains [63].

## 10. Conclusions

In this study, we tried to widen our understanding of the role of CRISPR-Cas9 on the treatment of autoimmune diseases through a comprehensive review of literatures. We also reviewed target genes and SNPs of various autoimmune diseases manipulated by the CRISPR-Cas9 method. The review of target genes and SNPs is organized in Table 1 and Table 2. As autoimmunity is based on an imbalance between pro-inflammatory and anti-inflammatory stimuli, cytokines such as IL1, IL36, and TNF alpha and T cell related factors are important in autoimmune diseases. By creating a synthetic sgRNA of a sequence complementary to a particular gene, Cas9, an RNA-guided DNA nuclease can knock out the genes related to the cytokines and T cell factors. Therefore, CRISPR-Cas9 may be a potential therapeutic approach to manage autoimmune diseases.

Recently, gene therapy and especially CRISPR-Cas9 method has become a promising approach as our understanding of the immunological and molecular basis of autoimmune diseases advances. Possibilities are not limited to frequently occurring diseases; it even holds great promise to treat rare diseases. Nevertheless, many of the studies on the treatment of autoimmune diseases using CRISPR-Cas9 have been conducted in cell studies so far and, therefore, more studies in humans are needed. Additionally, several technical challenges need to be addressed, such as off-target activity, insufficient indel or low homology-directed repair efficiency, in vivo delivery of the CRISPR-Cas9 system components, and immune responses [64]. Despite these limitations, the current evidence implies a promising role of CRISPR-Cas9 in the regulation of autoimmune diseases and further studies that focus on effectiveness of CRISPR-Cas9 method to human treatment are needed.

## Figures and Tables

**Table 1:** Human cell models using CRISPR-Cas9 in autoimmune diseases.

Study	Autoimmune Disease	Target Gene	SNP, miRNA	Results	**Ref**
Yang et al., 2020	RA	MYC, FOXO1	-	By collecting ATAC-seq, Hi-C, Capture Hi-C, and nuclear RNA-seq data in stimulated CD4+ T cells over 24 hours, the study provides evidence that MYC and FOXO1 genes may be causal factors of RA.	[42]
Yu et al., 2016	RA	IL20RA, IL22RA2, IFNGR1, OLIG3, TNFAIP3	SNP rs6927172	SNP rs6927172, TNFAIP3, and OLIG3 are importantly associated with the RA disease course.	[43]
Jing et al., 2015	RA	-	microRNA 155 (miR-155)	Genome editing of miR-155 can be a potential therapeutic strategy for RA.	[44]
Cardinale et al., 2020	IBD	-	SNP rs1887428	rs1887428 does not have very large impacts on JAK2 expression, but it impacts STAT5B downwards.	[45]
Mokhtar et al., 2019	IBD	SGK2	-	SGK2 protein was localized in cytoplasm of colonic epithelial cells both in long and short duration ulcerative colitis.	[46]
Li et al., 2018	IBD	PTPN2	SNP rs7234029	PTPN2 expression was increased in affected ileum compared to normal ileum and, in contrast, CRISPR-Cas9 mediated PTPN2 deletion resulted in higher levels of STAT3 and Erk1/2 phosphorylation and proliferation.	[47]
Matthews et al., 2019	IBD	-	SNP rs6651252	Using CRISPR-Cas9, they found that rs6651252 enhancer regulates expression of the c-MYC, and they also revealed that MYC expression levels are elevated in the Crohn’s disease patients.	[48]
Odqvist et al., 2019	SLE	TNFAIP3	SNP rs2230926	The result showed that A20 *C103A* cells or cells with rs2230926 polymorphism draw increased neutrophil extracellular trap and increased frequency of auto antibodies to citrullinated epitopes.	[53]
Harris et al., 2019	SLE	CXorf21	-	They have done in vitro CRISPR-Cas9 knockdown experiments and found that *CXorf21* knockdown resulted a decreased expression of TNF-alpha and IL-6.	[54]
Gregory et al., 2007	MS	IL7R	SNP rs6897932	There is a direct relationship between alternative rs6897932 alleles and MS risk.	[55]
Galarza-Munoz et al., 2017	MS	DDX39B	SNP rs2523506	It was shown that epistatic interaction between rs2523506 in *DDX39B* and rs6897932 in IL7R regulates IL7R exon 6 splicing and increases risk of MS.	[56]
Maier et al., 2009	MS	IL-2RA	-	IL2RA variants contributed to the risk of MS and T1D respectively.	[57]
Gregory et al., 2012	MS	TNFRSF1A	SNP rs1800693	TNF blocking agents have side effects that promote the onset of MS, when it is used in other autoimmune diseases such as RA, but this study found that a signal of disease association for rs1800693 is predictive of the side effect.	[58]
Zhu et al., 2019	T1DM	LCK	SNP rs10914542	They demonstrated that G allele of SNP rs10914542 of LCK increases the risk of Type 1 DM.	[59]
Arakawa et al., 2019	Psoriasis	ERAP1	-	They generated ERAP1 knockout melanoma cell line and revealed that epistasis between HLA-C*06:02 and ERAP1 variants will affect psoriasis.	[61]
Jouanin et al., 2019	Coeliac disease	α- or γ-gliadin	-	The result showed that it is possible to use CRISPR-Cas9 to edit α- or γ-gliadins and make safe forms of grains.	[63]

Abbreviations—SNP: single nucleotide polymorphism; miRNA: micro ribonucleic acid; RNA: ribonucleic acid; RA: rheumatoid arthritis; SNP: single nucleotide polymorphism; IBD: inflammatory bowel disease; CRISPR-Cas9: clustered regularly interspaced palindromic repeats and CRISPR-associated protein9; SLE: systemic lupus erythematosus; MS: multiple sclerosis; T1D: type 1 diabetes mellitus.

**Table 2:** Non-human cell models using CRISPR-Cas9 in autoimmune diseases.

Study	Autoimmune Disease	Target Gene	SNP, miRNA	Results	Ref
Pai et al., 2020	IBD	TL1A	-	The flare-up of TL1A, IFNγ, and MLCK is associated with IBD diseases course.	[49]
Friedrich et al., 2017	IBD	HDAC7	-	The result showed that HDAC function was reduced in IBD patients, and according to the knockout mice, HDAC were found to play a crucial role in maintenance of the intestinal barrier.	[50]
Eftychi et al., 2019	IBD	IFN-γ	-	They found out that many pro-inflammatory cytokines such as TNF, IL1b, and IL6 showed decreased expressivity in the KO mice and suggested that IFN-γ is essential for colon inflammation.	[51]
Ge et al., 2019	IBD	-	miRNA-125a	MicroRNA-125a suppresses Th1/Th17 cell differentiation and TNF-α production. Additionally, miR-125a knockout mice showed more severe forms of colitis.	[52]
Ratiu et al., 2017	T1DM	AID/RAD51	-	AID/RAD51 can be a target for treatment of Type 1 DM patients.	[60]
Roth-Carter et al., 2020	Psoriasis	Desmoglein 1	-	They supported a role of Dsg1 for differentiation of epidermis, formation of barriers, and regulation of inflammatory responses.	[62]

Abbreviations—IBD: inflammatory bowel disease; KO: knock out; T1DM: type 1 diabetes mellitus; Dsg1: desmoglein 1.
